# Supplementary material for: Social Touch Reduces Pain Perception—An fMRI Study of Cortical Mechanisms
Source: Brain Sci. 2023 Feb 24;13(3):393. doi: 10.3390/brainsci13030393 (PMC10046093; doi:10.3390/brainsci13030393)

Social touch reduces pain perception through mechanisms involving insula and periaqueductal gray

Mattias Savallampi[1], Anne M. S. Maallo[1], Sumaiya Shaikh[1], Francis McGlone[2], Frédérique Bariguian Revel[3], Håkan Olausson[1,4,5], Rebecca Boehme[1,5]*

1 Center for Social and Affective Neuroscience, Linköping University, Department of Biomedical and Clinical Sciences, 58185 Linköping, Sweden
2 Research Centre Brain & Behavior, Liverpool John Moores University, Liverpool, UK
3 GSK Consumer Healthcare S.A, Nyon, Switzerland
4 Department of Clinical Neurophysiology, Linköping University Hospital, 58185 Linköping, Sweden
5 Center for Medical Imaging and Visualization, Linköping University, 58185 Linköping, Sweden

Pressure device

The pressure device was custom-built and designed to reliably produce mechanical pressure and pain. The pneumatic part of the device (figure S1) was MR-safe, made out of hard plastic, and controlled via an air pump located in the control room and connected to a PC. The system has a maximum internal pressure of 5 PSI. The air pump inflates a cushion (from a blood pressure cuff, not depicted) in the bottom section of the device and thereby pushes up a peg (dark grey). The participant's hand is placed in the upper section of the device in such a way that the peg will press on the target region if activated. The conversion of internal air pressure to force at the head of the peg is linear, with a maximum of 60 N/ 1cm².

Figure S1: The MR-safe part of the pressure device. The hand is placed in the upper section and an inflatable pillow, connected to an air pump, is placed in the lower section, to move the peg up for mechanical pressure stimuli.

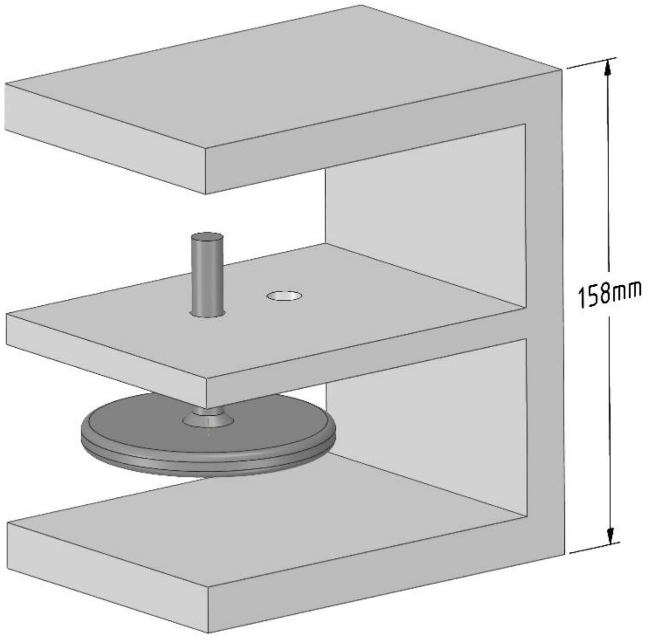

Supplement: Supplementary file 1 [file brainsci-13-00393-s001.zip › brainsci-2103635-supplementary.pdf]
